# Influence of inflammation on parasitism and area of experimental amoebic liver abscess: an immunohistochemical and morphometric study

**DOI:** 10.1186/1756-3305-4-27

**Published:** 2011-02-28

**Authors:** Cássia Abadia Xavier Costa, Thaisa Helena Silva Fonseca, Fabrício Marcus Silva Oliveira, Joseph Fabiano Guimaraes Santos, Maria Aparecida Gomes, Marcelo Vidigal Caliari

**Affiliations:** 1 Programa de Pós-Graduação em Patologia, Instituto de Ciências Biológicas da Universidade Federal de Minas Gerais. Av. Antônio Carlos 6627, Belo Horizonte, Minas Gerais, Brasil; 2 Departamento de Patologia Geral, Instituto de Ciências Biológicas da Universidade Federal de Minas Gerais. Av. Antônio Carlos 6627, Belo Horizonte, Minas Gerais, Brasil; 3 Departamento de Parasitologia, Instituto de Ciências Biológicas da Universidade Federal de Minas Gerais. Av. Antônio Carlos 6627, Belo Horizonte, Minas Gerais, Brasil; 4 Hospital Governador Israel Pinheiro - IPSEMG, Belo Horizonte, Minas Gerais, Brasil

## Abstract

The influence of inflammation on the number of trophozoites and on the murine amoebic liver abscess area following infection with *Entamoeba histolytica *and *E. dispar *was evaluated. Immunohistochemistry and digital morphometry were used to identify and quantify the trophozoites, neutrophils, macrophages, and lesions. Positive correlation was observed between the number of trophozoites and inflammatory cells. A significant decrease in parasitism and inflammation in groups treated with dexamethasone was observed. The scarceness or absence of trophozoites in the treated groups suggest the importance of the inflammatory response in the production of amoebic hepatic abscesses in spite of the inherent virulence of the parasite being decisive in the establishment of the lesion.

## Findings

Amoebiasis is a disease caused by the protozoan *Entamoeba histolytica *through oral infection by cysts followed by colonization or tissue invasion by trophozoites in the large intestine. Annually, about 50 million people are infected, causing approximately 100,000 deaths [1]. After the colonic mucosa is damaged, the trophozoites may reach the bloodstream and liver, producing the amoebic hepatic abscess, which is the most common extra-intestinal form of amoebiasis [2]. The typical amoebic lesion is characterized by a liquefactive necrosis zone with edges consisting of cellular debris and polymorph-histiocitary inflammatory infiltrate. Such necrosis is produced by trophozoite derivatives, such as amoebapores (polypeptides capable of forming pores in the hosts' cells), cysteine proteinases (enzymes that cleave collagen, elastin, fibrinogen, and laminin), and galactose/N-acetylgalactosamine lectin (Gal/GalNac) (a CD59 like molecule capable of inhibiting the C5b-9 complex of the complement, inducing apoptosis and stimulating the production of IL-1α, IL-1β, and IL-8).

Controversial results have been published about the role of inflammation on the pathogenesis of amoebic necrosis. The inflammatory infiltrate in amoebiasis is very discrete when compared to intense necrosis, suggesting that the inflammatory cells are destroyed, releasing their enzymatic content into the hepatic parenchyma [2]. Radiation-induced leucopenia in hamsters followed by trophozoites inoculation led to the reduction of inflammatory processes, hepatic necrosis, and parasitism [3]. On the other hand, mice infected with *E. histolytica *and previously treated with anti-neutrophil monoclonal antibodies developed liver necrosis with the same dimensions as those of the control group, and those lesions were probably produced by products secreted by the amoeba itself [4].

Considering the doubts that still persist about amoebic pathogenesis, we assessed the influence of inflammation in necrosis and in hepatic parasitism in mice inoculated with *E. histolytica*. For comparison purposes, we used the non-pathogenic species *E. dispar*, which can produce experimental lesions similar to those of *E. histolytica *and that has contributed to the understanding of the pathogenesis of amoebiasis [5,6]. Trophozoites from both strains, neutrophils, and macrophages were identified by immunohistochemistry and quantified by digital morphometry.

All procedures were conducted according to principles established by the Ethics Committee on Animal Experimentation of the UFMG. Ten Swiss mice were intra-hepatically inoculated with 250,000 trophozoites of the EGG strain of *E. histolytica *or MCR strain of *E. dispar*, previously identified by zymodeme and PCR [7,8]. Half of the animals were previously treated with two doses (0.5 mg/Kg) of dexamethasone, subcutaneously, 24 and 01 h before inoculation (Groups EGG-DEX and MCR-DEX) and, the other half received only buffered saline (Groups EGG and MCR).

The animals were euthanized on the second day of infection, three slices of the left lobe of the liver were collected, fixed in buffered formaldehyde 10%-pH 7.2, and scanned by a Sony MVC-CD400/CD250 digital camera for the morphometry of the amoebic necrosis. After dehydration, clearing and paraffin embedding of the slices, 4 μm thick cuts were obtained and stained with Haematoxylin and Eosin (H&E).

**Figure 1:**
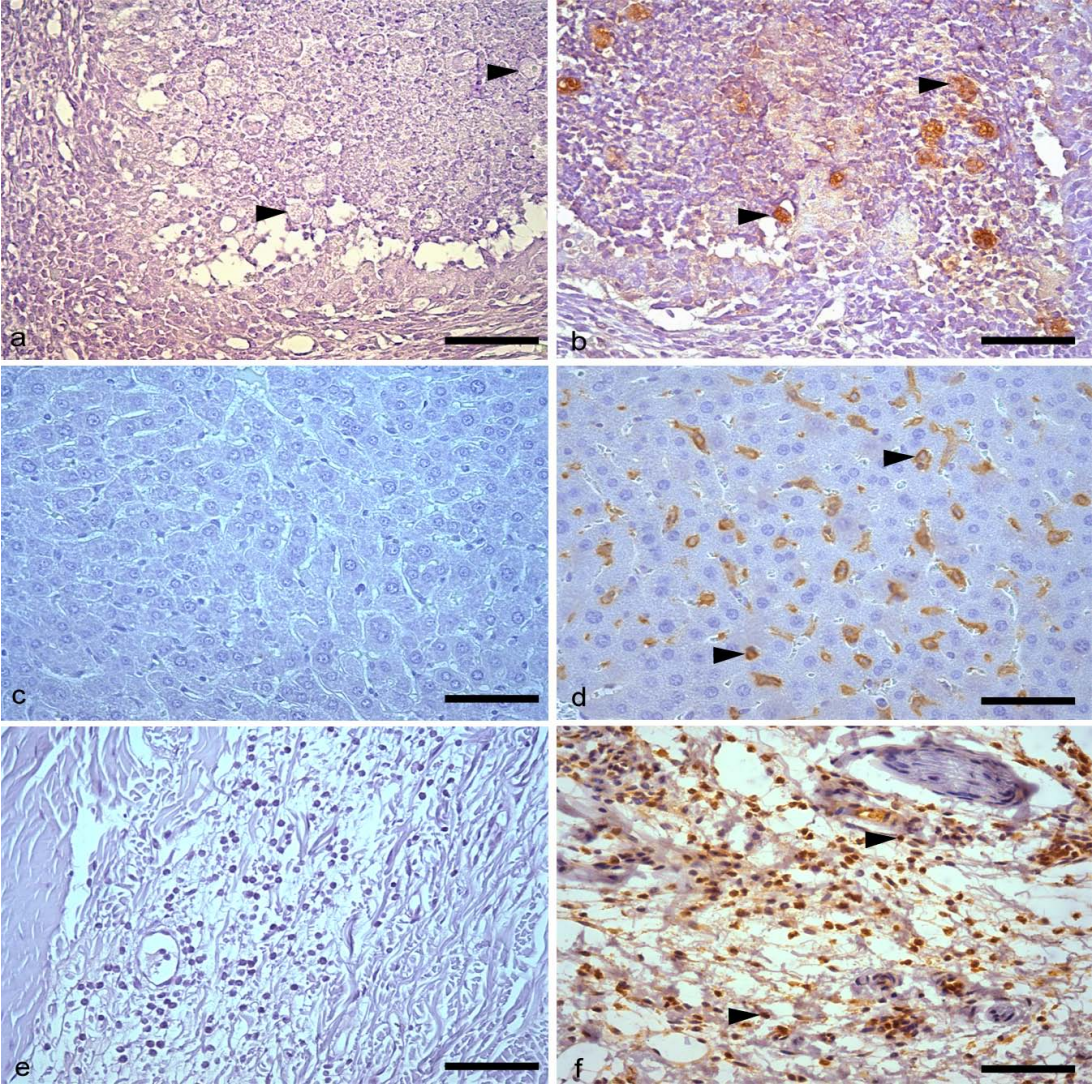
**Immunohistochemistry controls, positive (exposed to specific antibody) and negative (not exposed to specific antibody) from trophozoites, macrophages and neutrophils**. Hamster liver rich in trophozoites (arrowheads) in negative (a) and positive (b) controls for Immunohistochemical detection of trophozoites. Negative (c) and positive (d) macrophages (arrowheads) in mouse hepatic tissue. Negative (e) and positive (f) neutrophils (arrowheads) in mouse skin inflamed by intradermal injection of carrageenan. Bar 30 μm. Harris's haematoxylin counterstained.

Sections from the slices were washed in phosphate buffered saline (PBS)-pH 7.2, incubated in a H_2_O_2 _30vv-3.5% solution and goat serum. In order to identify neutrophils and macrophages, the sections were incubated with a Target-Retrieval (Dako-USA) solution at 100°C; incubated with anti-macrophage or anti-neutrophil monoclonal serum 1:50 (Hycult Biotechnology b.v.-Netherlands). In order to identify the trophozoites, the sections were incubated with anti-*E. histolytica *or anti-*E. dispar *polyclonal serum 1:1000 (*Laboratórios de Amebíases e Protozooses*/ICB/UFMG). All sections were incubated with biotinylated IgG and streptavidin conjugated with peroxidase 1:200 (Zymed Laboratories Inc., USA). In all reactions, the color was detected using a 0.05% diaminobenzidine solution in H_2_O_2_-40vv-0.2%; the sections were counterstained with Harris's haematoxylin. As a negative control, the primary antiserum was replaced by PBS in some sections (Figure 1). Positive controls consisted of sections of mouse skin inflamed by intradermal injection of carrageenan, and trophozoite-rich liver sections. Using Carl Zeiss-KS300 Software (Oberkochen, Germany), the necrotic areas of the liver slices were assessed.

Using a JVC-TK 1270 microcamera (JVC, Japan) and a 40× objective lens, 30 random images of immunohistochemical reactions against trophozoites were scanned, covering a total of 1.6 × 10^6 ^mm^2 ^analyzed hepatic parenchyma for the counting of trophozoites by that software. The macrophages and neutrophils were quantified on the edges of the necrosis using the same methodology. The distribution of continuous variables was assessed by the Shapiro-Wilk test, and this indicated normal distribution in all cases. Thus, the Student t test was used for comparison between groups, and differences with p < 0.05 were considered statistically significant. Data were expressed as mean ± standard deviation for parametric variables. The Pearson *r *was used for correlation analysis.

To exclude the possibility of amebicidal activity of dexamethasone, the growth inhibition of *E. histolytica *and *E. dispar *cultures associated with the drug was assessed. For this, 240,000 trophozoites were grown in 6 mL of medium at 37°C and associated with dexamethasone in concentrations ranging of 40 to 800 mg/mL for 48 hours. The tests were done in triplicate and repeated one time. There was no interference of drugs on the growth and development of cultures even in higher concentrations.

Hepatic amoebic lesions were detected in all animals inoculated with *E. histolytica *or *E. dispar*. Within 48 hours of infection, the lesion was macroscopically visible in the left lobe; it had a yellowish-white color and was well defined.

**Figure 2:**
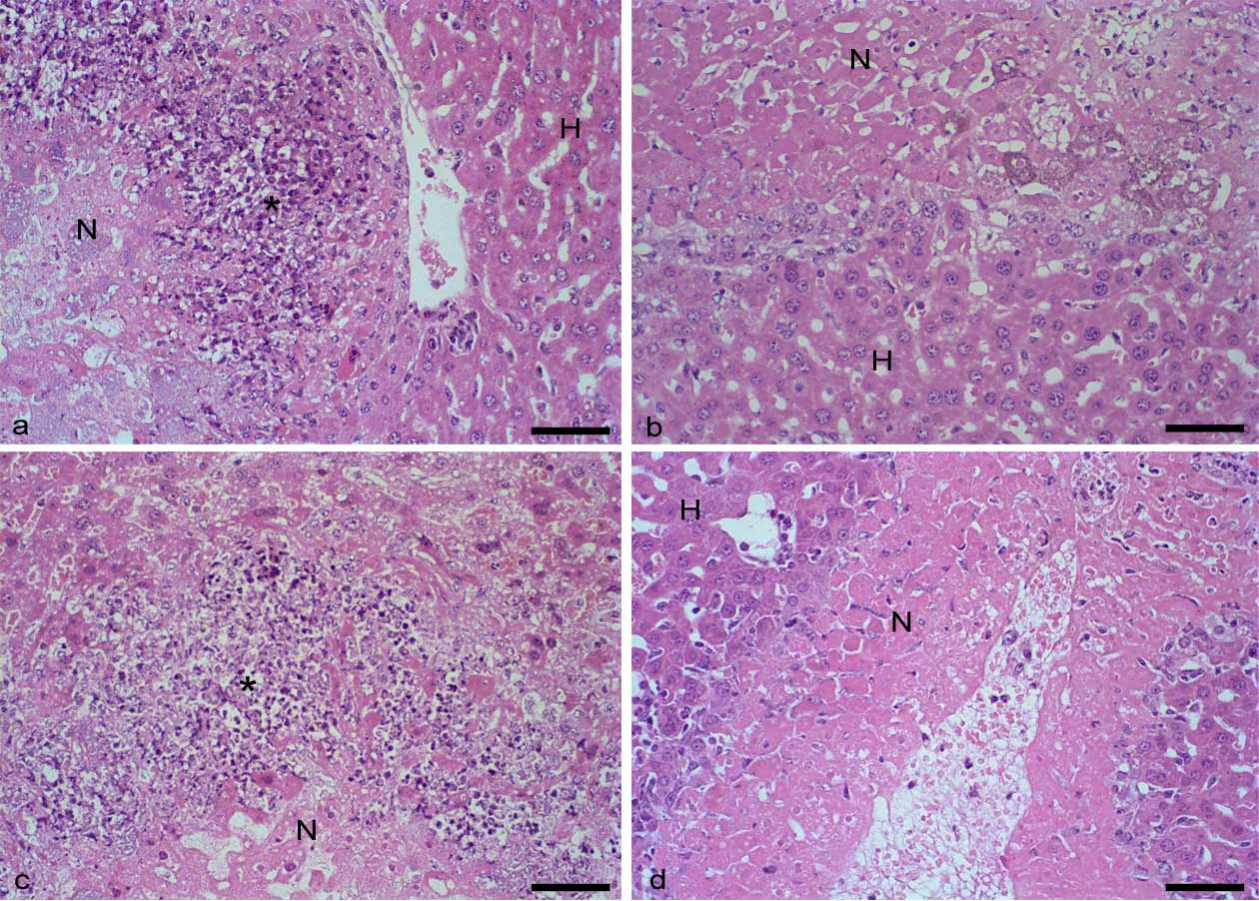
**Mouse liver inoculated with *E. histolytica *and *E. dispar *treated and untreated with dexamethasone**. Hepatic necrosis (N) induced in mice of the EGG group (a) and the MCR group (c). Debris and scarse inflammatory infiltrate (*). Non-necrotic hepatic parenchyma (H). EGG-DEX (b) and MCR-DEX (d) groups. Bar 50 μm. H&E.

Under the light microscope it was possible to observe the central area of liquefactive necrosis whose border consisted of cell debris, a discrete inflammatory infiltrate, and trophozoites. Polymorph-histiocitary inflammatory foci associated with cell debris and trophozoites were found in the control animals, as opposed to the animals treated with dexamethasone (Figure 2).

The mean and the standard deviation of the hepatic necrosis area in animals inoculated with *E. histolytica *of groups EGG and EGG-DEX were 2.95 ± 1.91 mm^2 ^and 1.32 ± 0.44 mm^2^, respectively. In animals inoculated with *E. dispar *of groups MCR and MCR-DEX, they were 4.63 ± 2.96 mm^2 ^and 2.62 ± 1.71 mm^2^, respectively. Although the mean area of necrosis proved to be arithmetically smaller in animals from both groups treated with dexamethasone, this difference between treatment groups was not significant by the Student's t-test (p > 0.05). The reduction of inflammation was associated with a small decrease in the necrotic area showing that necrosis in the untreated groups was mainly due to amoebic products. The secretion of amoebapores, cystein-proteinases, and Gal/GalNac lectin by the trophozoites also results in the destruction of neutrophils and the consequent liberation of their toxic products, which may play an important role in the amplification of amoebic lesions [2].

**Figure 3:**
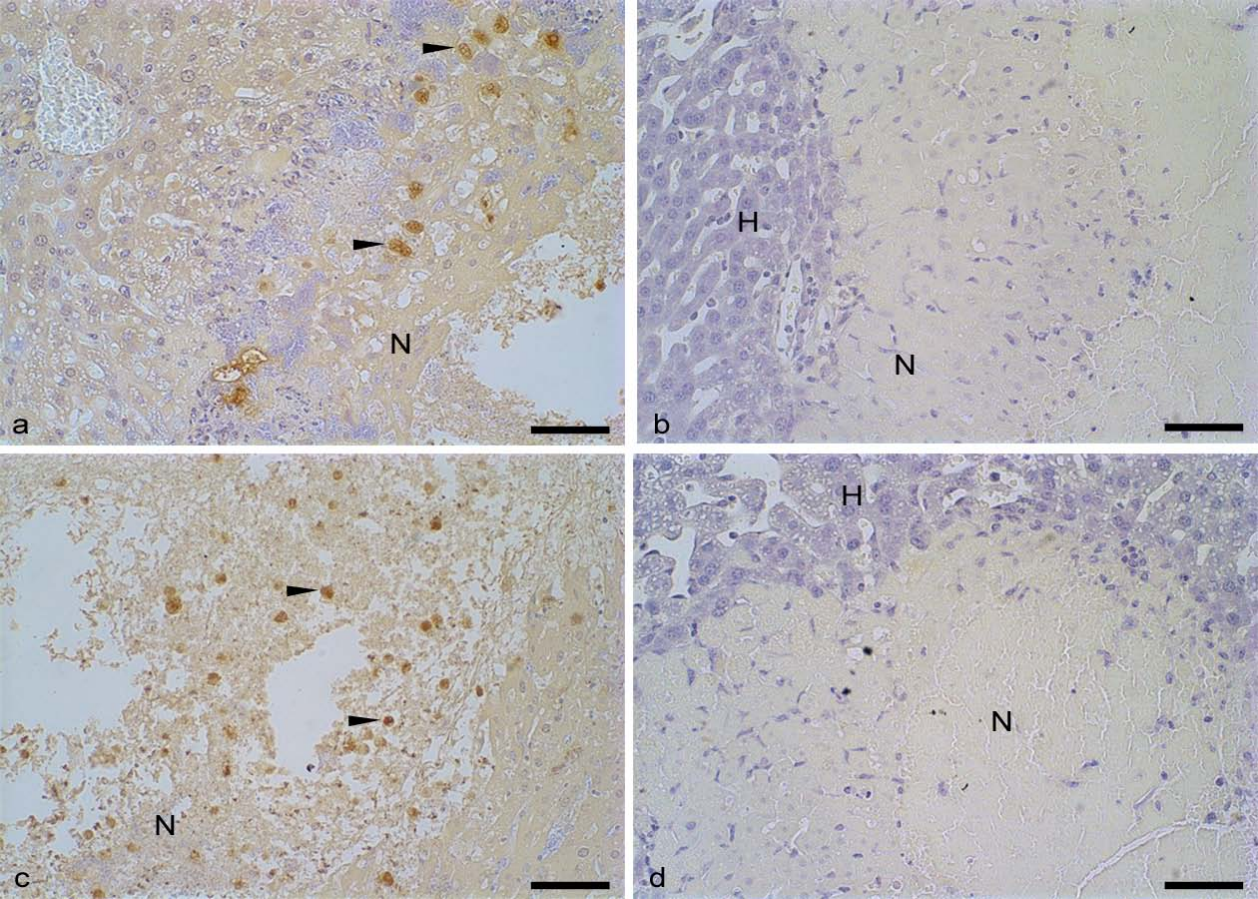
**Immunohistochemical reaction for trophozoites in the liver of mice inoculated with *E. histolytica *or *E. dispar *treated and untreated with dexamethasone**. Hepatic necrosis (N) induced in mice of the EGG group (a) and the MCR group (c). Trophozoites of *E. histolytica *and *E. dispar *immunostained within the hepatic necrosis (arrowheads). Absence of trophozoites in the hepatic necrosis (N) in the groups EGG-DEX (b) and MCR-DEX (d). Non-necrotic hepatic parenchyma (H). Bar 50 μm. Harris's haematoxylin counterstained.

**Figure 4:**
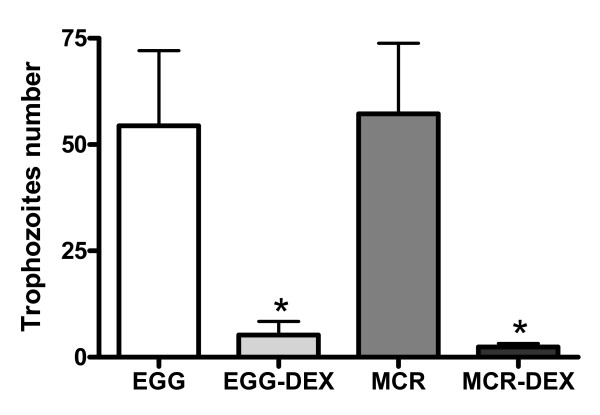
**Number of trophozoites in the hepatic parenchyma of mice inoculated with *E. histolytica *(EGG) or *E. dispar *(MCR), treated or not with dexamethasone**. * t = 2,73; p < 0.05

**Figure 5:**
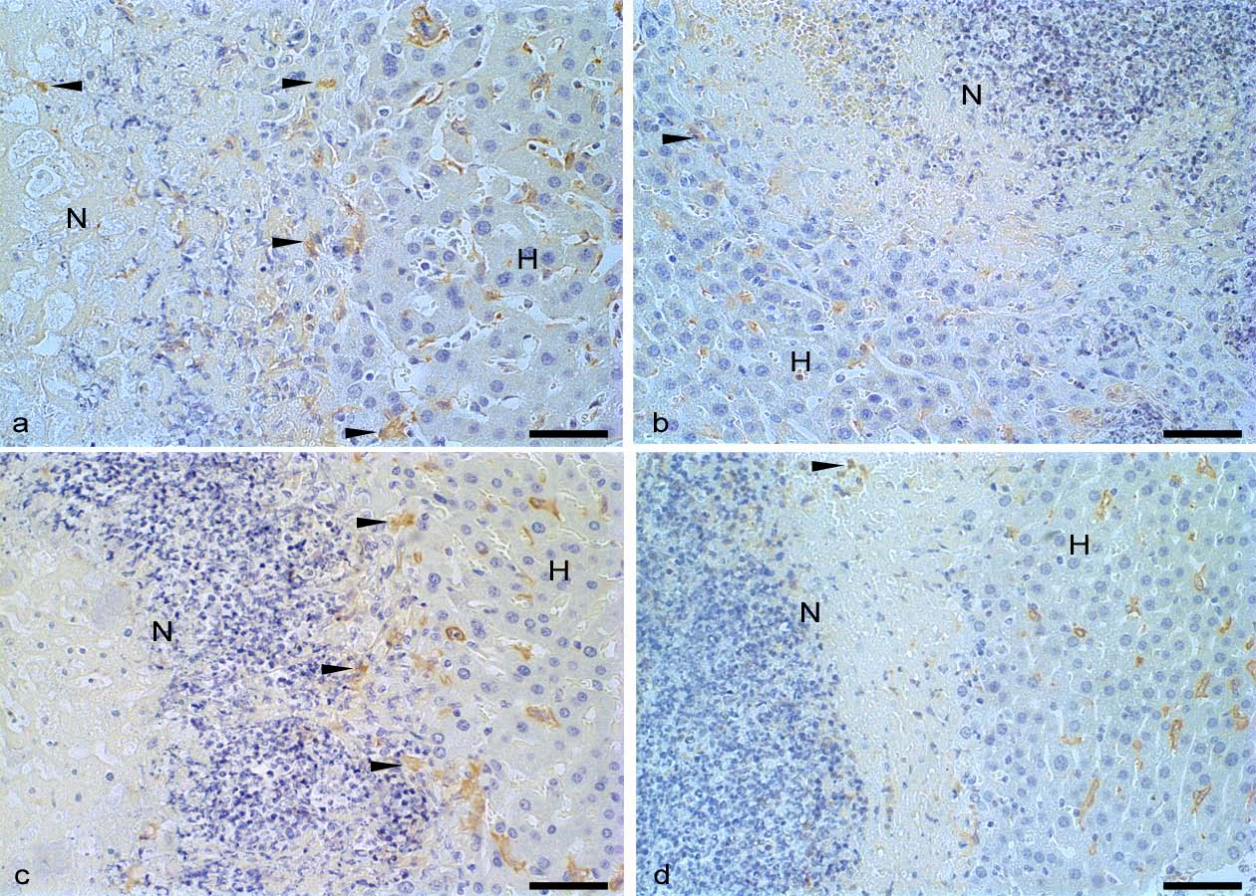
**Immunohistochemical reaction for macrophages in the liver of mice inoculated with *E. histolytica *or *E. dispar *treated and untreated with dexamethasone**. Scarcity of immunoreactive macrophages (arrowheads) adjacent to the debris-rich hepatic necrosis (N) in the groups EGG-DEX (b) and MCR-DEX (d) compared to groups EGG (a) and MCR (c). Non-necrotic hepatic parenchyma (H). Bar 50 μm. Harris's haematoxylin counterstained.

**Figure 6:**
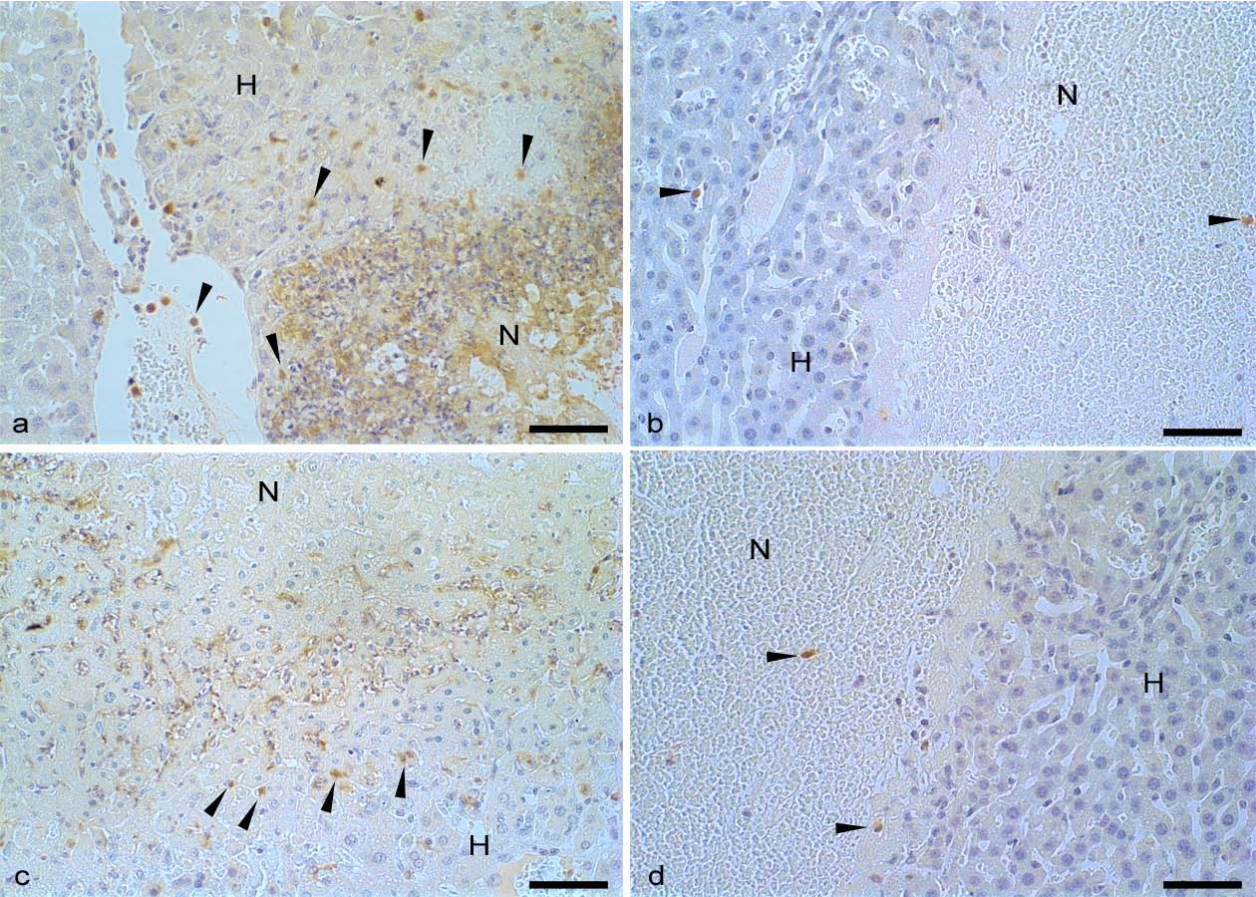
**Immunohistochemical reaction for neutrophils in the liver of mice inoculated with *E. histolytica *or *E. dispar *treated and untreated with dexamethasone**. Increased number of immunoreactive neutrophils (arrowheads) adjacent to hepatic necrosis (N) in the groups EGG (a) and MCR (c) compared to groups EGG-DEX (b) and MCR-DEX (d). Non-necrotic hepatic parenchyma (H). Bar 50 μm. Harris's haematoxylin counterstained.

Immunohistochemistry revealed the presence of trophozoites mainly on the edges of the central zone of necrosis, as well as inside it, in the hepatic sinusoids, and in inflammatory foci. The quantitative analysis showed a significant decrease in the number of trophozoites and of inflammatory foci in the animals in groups EGG-DEX and MCR-DEX (Figures 3 and 4) (p < 0.05). In groups EGG and MCR, immunohistochemistry showed a predominance of macrophages in the inflammatory infiltrate, not only on the edges of the necrosis zone but also in the inflammatory foci (Figure 5), whereas the neutrophils were found in small quantities in the same areas (Figure 6). *In vitro *studies showed that macrophages activated after stimulation via IFN-gamma showed greater resistance and ability to destroy trophozoites through NO production [9]. When stimulated with amoebic proteins, macrophages isolated from hepatic abscesses in the initial stages of infection produced large concentrations of TNF-a [10]. The need for macrophage activation suggests a secretory Th1 response of IFN-gamma, IL-2, and TNF-alpha. On the other hand, in experimental amoebic colitis the same intensity of parasitism and inflammation was observed in animals deficient in IL-2, IFN-gamma, and NO [11].

The quantitative analysis showed a significant decrease of macrophages on the edges of the necrosis zone of the animals in the MCR-DEX group (p < 0.05). The mean and the standard deviation of the number of macrophages in animals inoculated with *E. dispar *from groups MCR and MCR-DEX were 23 ± 8.91 and 5.8 ± 5.67, respectively. A positive correlation was observed between the fall in the number of trophozoites and the reduction in the number of macrophages in the necrotic zone (r = 0.6217; p = 0.0017) and the reduction in the number of neutrophils in the hepatic parenchyma (r = 0.3939; p = 0.04). In groups EGG (12.4 ± 3.4) and EGG-DEX (9.2 ± 8.2) there was no significant reduction in the number of macrophages at the edge of the lesion.

As expected, the treatment with dexamethasone reduced the inflammatory infiltrate, but, paradoxically, the number of trophozoites and necrotic area were smaller. Such results suggest that the inflammation normally observed during the amoebic pathogenesis favors the multiplication of trophozoites and, consequently, leads to an increase in the dimensions of the amoebic abscess area. It is possible that leukocytes and/or their products stimulate the transcription of amoebic toxic substances, amplifying the lesion. Additionally, a study performed with trophozoites marked with radioactivity showed that less resistant trophozoites were destroyed by inflammation, favoring the proliferation of more virulent parasites [12]. This probable selection of parasites by the inflammatory process has also been indicated by our studies with the experimental model of amoebic liver abscess in hamster [13,14]. In animals inoculated with either *E. histolytica *or *E. dispar *small numbers of trophozoites were found in the liver parenchyma after 12 hours of infection. However, within 24 hours of infection a significant increase of trophozoites was observed in mice infected with both species of amoebae (7 and 130 times in *E. histolytica *and *E. dispar*, respectively), showing that, although both produce experimental lesions, their mechanisms of adaptation to the host are different. Perhaps the lowest number of trophozoites observed in *E. histolytica *infection would be related to its higher virulence. *E. histolytica *produces greater amount and variety of proteolytic substances, leading not only to the destruction of host tissue but also of amoebae. No significant reduction observed in the number of macrophages at the edge of the lesion in the EGG group leads us to speculate that these cells are not major factors involved in virulence selection on *E. histolytica*.

The visualization of residual trophozoites with positive immunohistochemical staining for immunoglobulins and complement, suggests that these elements may be involved in the selection of more resistant parasites [14]. Genotype differences from *E. histolytica *and *E. dispar *in stool- and liver abscess-derived samples from the same patients [15,16] corroborate the selection hypothesis.

In spite of the earlier published controversial results about the importance of the inflammatory response in the production of amoebic hepatic abscesses, our results emphasise the crucial role of inflammation in pathogenesis and establishment of the lesion.

## Competing interests

The authors declare that they have no competing interests.

## Authors' contributions

CCAX, GMA and CMV conceived the study. CCAX, GMA and FTHS by rearing the amoebas, made the inocula, liver inoculation of hamsters and in vitro tests. CCAX, OFMS and CMV made the necropsy of mice, histopathological procedures and morphometry analysis. CCAX, OFMS and FTHS made the immunohistochemical assays. SJFG made the statistical analysis. All authors participated in the study design, analysis of the results, drafted the manuscript and have given final approval of the version to be published.
